# Microbial feedback drives soil carbon fixation and nutrient transformation during *Euphorbia jolkinii* expansion in subalpine meadows

**DOI:** 10.3389/fmicb.2026.1864391

**Published:** 2026-07-15

**Authors:** Lai-Xiang Ma, Xue Xiao, Qiong-Mei Niu, Jian-Gui Zhang, Zhi-Li Zhao, Yang Yang, Jia-Qi Lu, Xiao-Hui Chu, Gui-Lian Shan

**Affiliations:** Faculty of Animal Science and Technology, Yunnan Agricultural University, Kunming, China

**Keywords:** carbon transformation, *Euphorbia jolkinii*, native plant expansion, nutrient availability, soil microecological environment

## Abstract

**Background and aims:**

*Euphorbia jolkinii (E. jolkinii)*, a native toxic weed widely occurring in the subalpine meadows of the Southern Tibetan Plateau, has increasingly expanded in recent years, leading to reduced forage availability and potential livestock poisoning. Although its ecological impacts are evident, the mechanisms supporting its spread remain inadequately clarified. In particular, how expansion intensity interacts with soil microorganisms and carbon transformation processes requires further exploration.

**Methods:**

Field patches exhibiting different levels of *E. jolkinii* expansion were selected as the research subjects. Across different expansion levels, variations in soil carbon fractions, microbial community composition and diversity, as well as functional genes involved in the carbon cycle, were systematically analyzed.

**Results:**

The expansion of *E. jolkinii* was associated with a significant decrease in soil pH along with concurrent increases in the contents of available nitrogen (AN), available phosphorus (AP), total nitrogen (TN), total phosphorus (TP), and available potassium (AK). Expanded patches exhibited a distinct microecological environment characterized by the enrichment of key microbial taxa, particularly *Bradyrhizobium* and *Bacillus*. These microbial groups were coupled with shifts in soil carbon fixation and organic carbon turnover potential, corresponding to significantly higher contents of soil organic carbon (SOC), easily oxidizable organic carbon (EOC), microbial biomass carbon (MBC), and dissolved organic carbon (DOC).

**Conclusion:**

*Euphorbia jolkinii* expansion is closely associated with the enrichment of soil microbial guilds involved in carbon cycling. The concurrent enhancement in soil carbon fixation and transformation potential corresponds to altered nutrient availability, suggesting a potential microbial feedback mechanism that aligns with the successful encroachment of this species in subalpine meadows.

## 1 Introduction

Grasslands represent one of the most extensive terrestrial ecosystems worldwide and are fundamental to ecological stability as well as regional economic activities (Zou et al., 2022). In recent years, however, subalpine meadows on the Tibetan Plateau have shown clear signs of degradation. This trend is largely associated with both environmental pressures, such as climate variability, and human disturbances, particularly overgrazing. Under these combined influences, several toxic weeds, including *Euphorbia jolkinii (E. jolkinii)*, *Stellera chamaejasme* (*S. chamaejasme*), and *Ligularia virgaurea* (*L. virgaurea*), have expanded rapidly. Similar patterns have also been observed in the subalpine meadows of Northwestern Yunnan, located along the Southern Tibetan Plateau. The spread of these toxic weeds threats the sustainability of grassland-based animal husbandry in Tibetan Plateau region (Chen et al., 2024).

Effective management of poisonous weed expansion is essential for the restoration and sustainable management of degraded grasslands on the Southern Tibetan Plateau, while a clear understanding of the mechanisms underlying their expansion provides the foundation for developing effective control strategies (Li et al., 2024). As a fundamental element required for plant growth and metabolism, carbon plays a central role in the expansion of poisonous weeds through shifts in carbon cycling processes (Hou et al., 2024). One important mechanism underlying the expansion of poisonous weeds involves the regulation of carbon “uptake-transformation-allocation-storage” continuum, enabling these species to gain a competitive advantage in carbon resources. At the same time, interactions between carbon cycling and other nutrient processes contribute to the formation of soil microenvironments that favor their persistence and spread (Qin et al., 2022). Soil microorganisms are deeply embedded in these processes, as they mediate carbon fixation, decomposition, and methane-related pathways (Liang and Zhu, 2021; Zheng et al., 2022). Previous studies suggested that *S. chamaejasme* can reshape microbial communities through root exudates, thereby increasing the availability of carbon for its own growth (Zhang et al., 2024). Likewise, *L. virgaurea* has been reported to modify soil carbon cycle indirectly by altering microbial community composition and structure (Song et al., 2023).

*Euphorbia jolkinii*, a perennial poisonous species of the Euphorbiaceae family, is widely recognized as one of the dominant native toxic weeds in subalpine meadows along the Southern Tibetan Plateau (Xiao W. et al., 2025; Xiao X. et al., 2025; Xiao Y. et al., 2025). Its expansion is frequently accompanied by a decline in palatable forage species, which is partly attributed to its strong capacity for nutrient enrichment. Field observations have shown that expanded patches tend to accumulate higher levels of litter carbon, soil organic carbon (SOC), and labile carbon fractions. In parallel, key microbial taxa associated with carbon cycle, such as *Edaphobacter*, often increase in relative abundance (Yang et al., 2025). However, the relationships between *E. jolkinii*-induced shifts in soil microbial community structure and the processes of soil carbon fixation and transformation remain insufficiently understood.

To address this gap, subalpine meadows systems in Shangri-La on the Southern Tibetan Plateau were selected as the study area. Given the patchy distribution of *E. jolkinii*, sites were categorized into non-expanded (N), lightly expanded (L), and heavily expanded (H) patches to capture different expansion stages. Soil samples from these patches were analyzed for physicochemical properties and carbon fractions, while metagenomic approaches were used to characterize functional microbial groups and genes involved in key carbon cycle processes. By integrating these datasets, this study aims to clarify how changes in microbial community structure relate to soil carbon transformation under *E. jolkinii* expansion, and to further explain its expansion from the perspective of microbially mediated nutrient cycle.

## 2 Methods

### 2.1 Study site

The study site was located in Wadi Village Group, Heping Community, Xiaozhongdian Town, Shangri-La City, situated along the southern margin of the Tibet Plateau (27°53′77″N, 99°82′37″E; 3,191 m a.s.l.). The site covers an area of approximately 17.65 ha. Vegetation at the site is typical of subalpine meadow ecosystems and is primarily dominated by *Poa crymophila (P. crymophila)*, a cold-tolerant and early-growing grass species. Within this community, *E. jolkinii* occurs as a native toxic weed and shows a clear patchy distribution pattern across the meadow. The soil is classified as mountain meadow soil. Climatic conditions correspond to a cold-temperate montane monsoon regime. The mean annual temperature is 5.8 °C, with an accumulated temperature (≥10 °C) of 2006.9 °C. Annual precipitation averages 647 mm, and the site receives about 2,168 h of sunshine each year. Relative humidity remains around 70%, and the frost-free period lasts approximately 244 d. The area is also characterized by pronounced diurnal temperature fluctuations and strong solar radiation. The grassland is currently managed under a free-grazing system. Grazing pressure is relatively moderate, corresponding to approximately 3 Tibetan pigs·ha^−1^ and 0.5 yaks·ha^−1^.

### 2.2 Experimental design and sample collection

**Figure 1:**
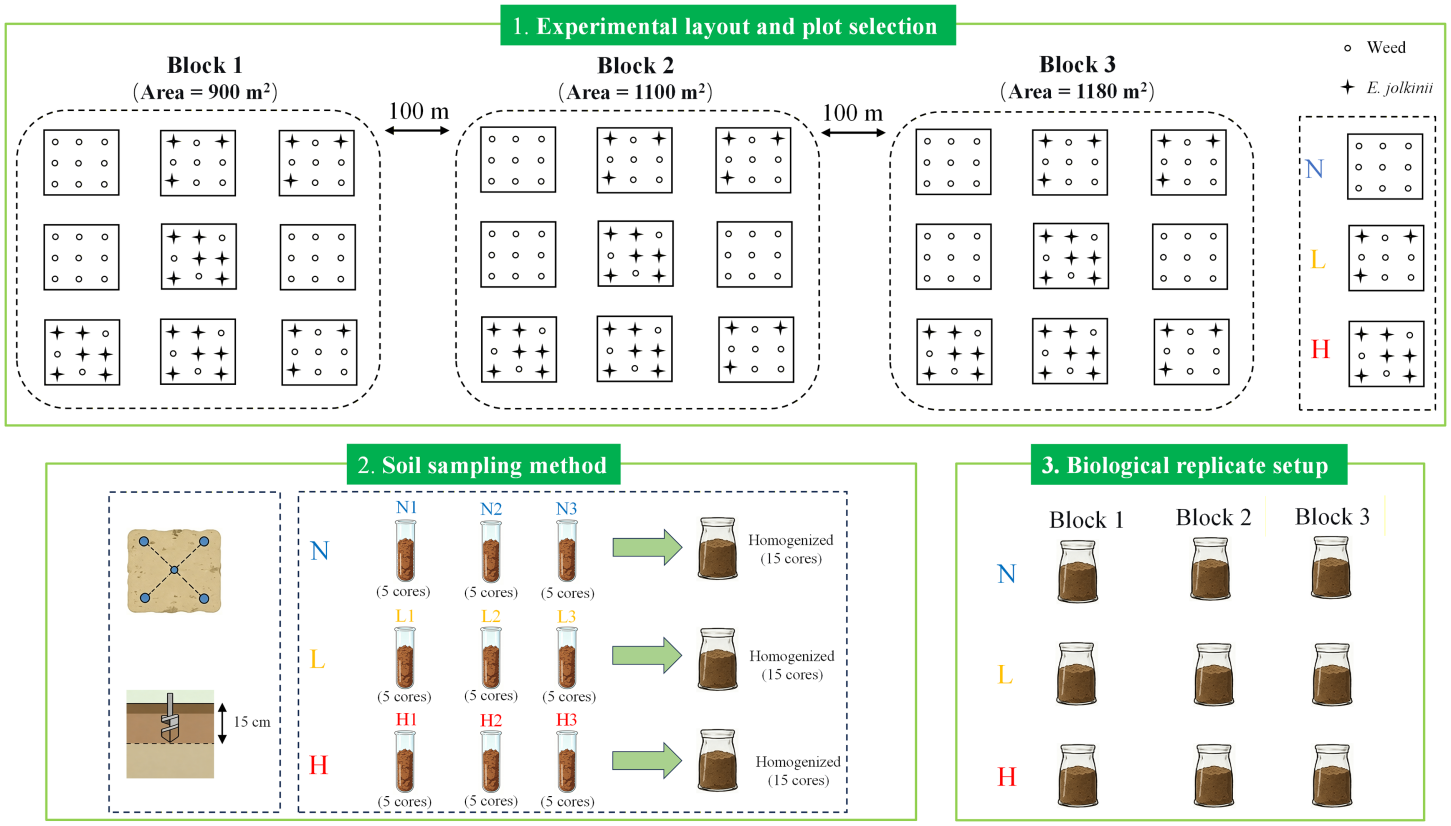
Schematic diagram of the experimental layout and soil sampling workflow. Three patch types were selected within each block based on the coverage of *E. jolkinii*: N, non-expanded patches; L, lightly expanded patches; and H, heavily expanded patches. The detailed sampling method and biological replicate setup (homogenization of 15 soil cores per composite sample) are illustrated in panels 2 and 3.

This experiment was conducted using a randomized complete block design. Three experimental blocks with areas of 900 m^2^, 1,100 m^2^, and 1,180 m^2^ were set up in the study plot, with a 100 m interval between adjacent blocks (Figure 1). Given the patchy spatial distribution of *E. jolkinii* following its expansion, three patch types were established in each block in accordance with the patch classification criteria proposed by Xiao et al. (2026): non-expanded patches (N), lightly expanded patches (L, *E. jolkinii* coverage <10%), and heavily expanded patches (H, *E. jolkinii* coverage >40%). The basic characteristics of the three patch types are detailed in Supplementary Table 1. To ensure representativeness and uniformity of sampling, three replicate sampling units were deployed for each patch type within every block, yielding a total of nine sampling units per patch type across all blocks.

In mid-August 2024, soil sampling was performed using the five-point sampling method. The sampling depth was set at 0–15 cm, as non-expanded patches are dominated by shallow-rooted fibrous gramineous forages, and this soil layer serves as the primary horizon for organic matter accumulation and microbial activity in meadow soils. For each sampling unit, five sampling points were established, and one soil core was retrieved from each point. Fifteen soil cores collected from the three sampling units of the same patch type within a single block were thoroughly mixed in equal proportions to generate a composite sample, representing one biological replicate. Accordingly, each patch type obtained three replicates across the three experimental blocks. All soil samples were immediately transported to the laboratory in portable refrigerated containers and sieved through a 2 mm mesh. The processed samples were divided into three portions: one was air-dried for the determination of soil physicochemical properties, one was stored at 4 °C for microbial biomass carbon (MBC) analysis, and the remaining one was preserved at −80 °C for subsequent metagenomic sequencing.

### 2.3 Determination of soil physicochemical properties

Soil pH was measured in a 1:5 (w/v) soil-water suspension using a pH meter (Mettler-Toledo GmbH, Greifensee, Switzerland). Nitrogen-related indices were assessed using standard procedures: total nitrogen (TN) was analyzed via the Kjeldahl method, whereas available nitrogen (AN) was determined using the alkali hydrolysis diffusion approach. Phosphorus fractions, including total phosphorus (TP) and available phosphorus (AP), were quantified using the molybdenum-antimony colorimetric method. Potassium content was evaluated by flame photometry, covering both total potassium (TK) and available potassium (AK). For carbon components, SOC and dissolved organic carbon (DOC) were both quantified using the potassium dichromate oxidation-volumetric method with external heating. Easily oxidizable organic carbon (EOC) was analyzed using potassium permanganate oxidation with colorimetric detection. MBC was determined following the chloroform fumigation-extraction procedure (Bao, 2000).

### 2.4 Soil microorganism sequencing and examination

Soil DNA extraction and quality assessment followed the protocol described by Zhu et al. (2024). Briefly, DNA was isolated from 0.50 g of soil using the PowerSoil® DNA Isolation Kit (MOBIO, USA). DNA quality was subsequently checked by 1% agarose gel electrophoresis for integrity, while purity was evaluated using a nucleic acid quantifier to confirm suitability for downstream sequencing. Metagenomic sequencing was carried out on the Illumina NovaSeq X Plus platform (PE150; Illumina Inc., San Diego, CA, USA). Library preparation involved end repair, 3′-A tailing, and adapter ligation, followed by magnetic bead purification and fragment size selection to remove self-ligated products. The final libraries were obtained after polymerase chain reaction (PCR) amplification. Sequencing, as well as downstream read assembly and gene annotation, was completed by Guangzhou Gidio Biotechnology Co., Ltd. Raw reads were first subjected to quality control using FastQC (v0.11.9). Sequences shorter than 200 bp, containing ambiguous bases, or with an average quality score below 20 were discarded. The remaining high-quality reads were assembled for each sample to generate contigs and scaffolds. Open reading frames (ORFs) were predicted from assembled contigs using MetaGeneMark, which is specifically designed for prokaryotic and metagenomic datasets. The resulting gene and protein sequences were further processed to construct a non-redundant dataset. For this step, MMseqs2 was applied with a similarity threshold of 95% and a minimum alignment coverage of 90% (based on the shorter sequence). Functional annotation was conducted by comparing the non-redundant sequences against the Kyoto Encyclopedia of Genes and Genomes (KEGG) database. Gene abundance in each sample was estimated by mapping clean reads back to the annotated gene set using CD-HIT (v2.1.0). Based on KEGG pathway assignments, genes involved in the carbon cycle were identified and grouped into functional categories, including carbon fixation, carbon decomposition, and methane metabolism.

### 2.5 Statistical evaluation

Statistical evaluation was primarily conducted using SPSS Statistics 26 (IBM, USA). Differences among treatments were evaluated through one-way analysis of variance (ANOVA), followed by Duncan’s multiple range test where appropriate. Data visualization was carried out in Origin 21.0, including boxplots, bar charts, correlation heatmaps, and pie charts. Microbial diversity analyses were implemented in R. The *α*-diversity of bacterial and fungal communities was estimated using the “vegan” package, whereas *β*-diversity was assessed based on Bray–Curtis dissimilarity and further explored through principal coordinate analysis (PCoA). To further identify key drivers, random forest models were developed in R (version 4.3.0). Additionally, partial least squares path modeling (PLS-PM) was employed to examine the interrelationships among soil pH, microbial communities, environmental factors, key genes involved in carbon fixation and decomposition, and soil carbon fractions (EOC, MBC, DOC, and SOC) (Yang et al., 2024). Model construction and visualization were performed using the “plspm” and “vegan” packages in R. The significance of directional coefficients (path coefficients) was estimated based on the default standard errors of the model, and overall model performance was evaluated using the goodness-of-fit (GOF) index.

## 3 Results

### 3.1 Impact of *Euphorbia jolkinii* expansion on soil physicochemical properties

The *E. jolkinii* expansion was accompanied by pronounced changes in soil environmental conditions within subalpine meadows on the Southern Tibetan Plateau. A consistent decline in soil pH was observed in both L and H patches compared with N (*p* < 0.05). In contrast, several nutrient-related variables showed clear increases following expansion. Specifically, TN, AP, SOC, EOC, and MBC were all elevated in expanded patches (*p* < 0.05). Some indicators, including AN, TP, AK, and DOC, exhibited significant increases only in H patches (*p* < 0.05). By comparison, TK remained relatively stable and did not differ significantly among patch types (Table 1).

**Table 1:** Variation in soil physicochemical properties among various patch types.

Physicochemical properties	N	L	H
pH	5.70 ± 0.03a	5.59 ± 0.01b	5.44 ± 0.01c
TN (g·kg^−1^)	5.36 ± 0.01c	6.34 ± 0.03b	6.63 ± 0.01a
TP (g·kg^−1^)	1.45 ± 0.02b	1.46 ± 0.01b	1.56 ± 0.01a
TK (g·kg^−1^)	12.33 ± 0.15a	12.30 ± 0.17a	12.57 ± 0.03a
AN (mg·kg^−1^)	547.00 ± 5.86b	549.67 ± 12.24ab	575.67 ± 1.20a
AP(mg·kg^−1^)	7.35 ± 0.15c	10.56 ± 0.29b	12.66 ± 0.33a
AK (mg·kg^−1^)	198.00 ± 1.53b	194.00 ± 0.58b	251.67 ± 2.85a
SOC (g·kg^−1^)	53.07 ± 1.20b	64.90 ± 0.90a	68.70 ± 2.95a
EOC (g·kg^−1^)	0.13 ± 0.05b	0.30 ± 0.03a	0.29 ± 0.04a
MBC (mg·kg^−1^)	614.78 ± 5.17c	749.63 ± 13.47b	838.00 ± 23.64a
DOC (mg·kg^−1^)	408.87 ± 8.39b	427.25 ± 10.86b	530.58 ± 8.96a

Data are expressed as mean ± standard error. Different lowercase letters (a, b, c) indicate significant differences among patches within the same line (*p* < 0.05). N: Soil in non-expanded patches by *E. jolkinii*; L: Soil in lightly expanded patches by *E. jolkinii*; H: Soil in heavily expanded patches by *E. jolkinii*. TN, TP, TK, AN, AP, AK, SOC, EOC, MBC, and DOC represent total nitrogen, total phosphorus, total potassium, available nitrogen, available phosphorus, available potassium, soil organic carbon, easily oxidizable organic carbon, microbial biomass carbon, and dissolved organic carbon, respectively; the same as below.

### 3.2 Impact of *Euphorbia jolkinii* expansion on soil microbial community structure

**Figure 2:**
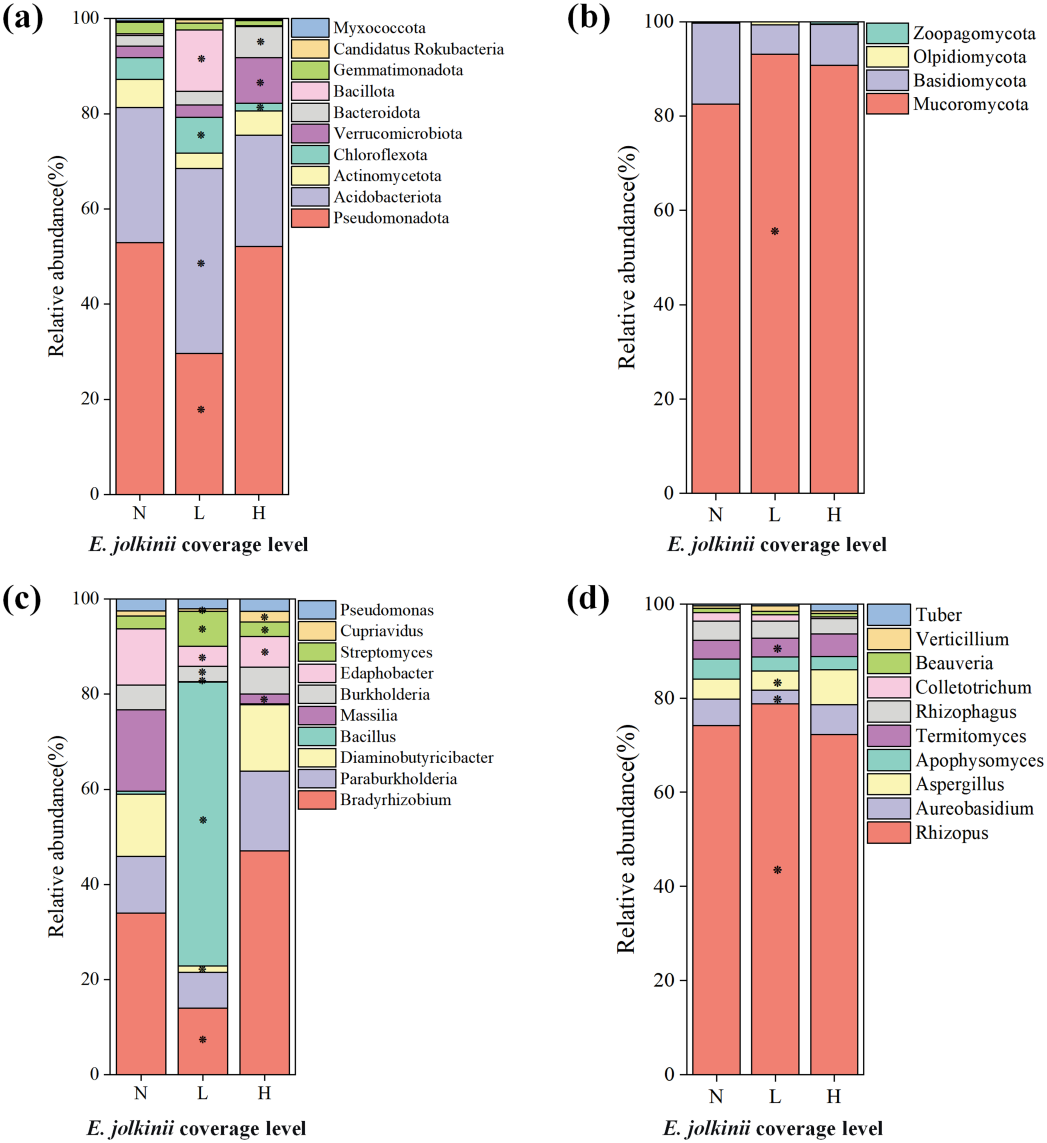
Soil microbial communities in patches with various invasion statuses: **(a)** Bacterial community composition at phylum level; **(b)** Fungal community composition at phylum level; **(c)** Bacterial community composition at genus level; **(d)** Fungal community composition at genus level (Significant differences are indicated by “⁕”). N: Soil in non-expanded patches by *E. jolkinii*; L: Soil in lightly expanded patches by *E. jolkinii*; H: Soil in heavily expanded patches by *E. jolkinii*.

Soil microbial communities in subalpine meadows on the Southern Tibetan Plateau were mainly composed of Pseudomonadota and Acidobacteriota for bacteria, whereas fungi were largely dominated by Mucoromycota. Compared with N patches, the relative abundance of Pseudomonadota decreased significantly in L patches, whereas the abundances of Acidobacteriota, Chloroflexota, and Bacillota increased significantly (*p <* 0.05; Figure 2a). In H patches, the relative abundance of Chloroflexota declined significantly, while Verrucomicrobiota and Bacteroidota showed significant increases (*p <* 0.05; Figure 2a). The abundance of Mucoromycota also increased following expansion; however, a statistically significant difference was observed only between L and N patches (*p <* 0.05; Figure 2b).

At a finer taxonomic resolution, distinct patterns emerged at the genus level (Figures 2c,d). In bacterial communities, L patches exhibited significantly lower abundances of *Bradyrhizobium*, *Diaminobutyricibacter*, *Massilia*, *Burkholderia*, *Edaphobacter*, and *Cupriavidus* compared with N patches, whereas the abundances of *Bacillus* and *Streptomyces* increased significantly (*p <* 0.05; Figure 2c). In H patches, the abundances of *Massilia* and *Edaphobacter* declined significantly, while *Streptomyces* and *Cupriavidus* became significantly enriched (*p <* 0.05; Figure 2d).

### 3.3 Impact of *Euphorbia jolkinii* expansion on soil microbial diversity

**Figure 3:**
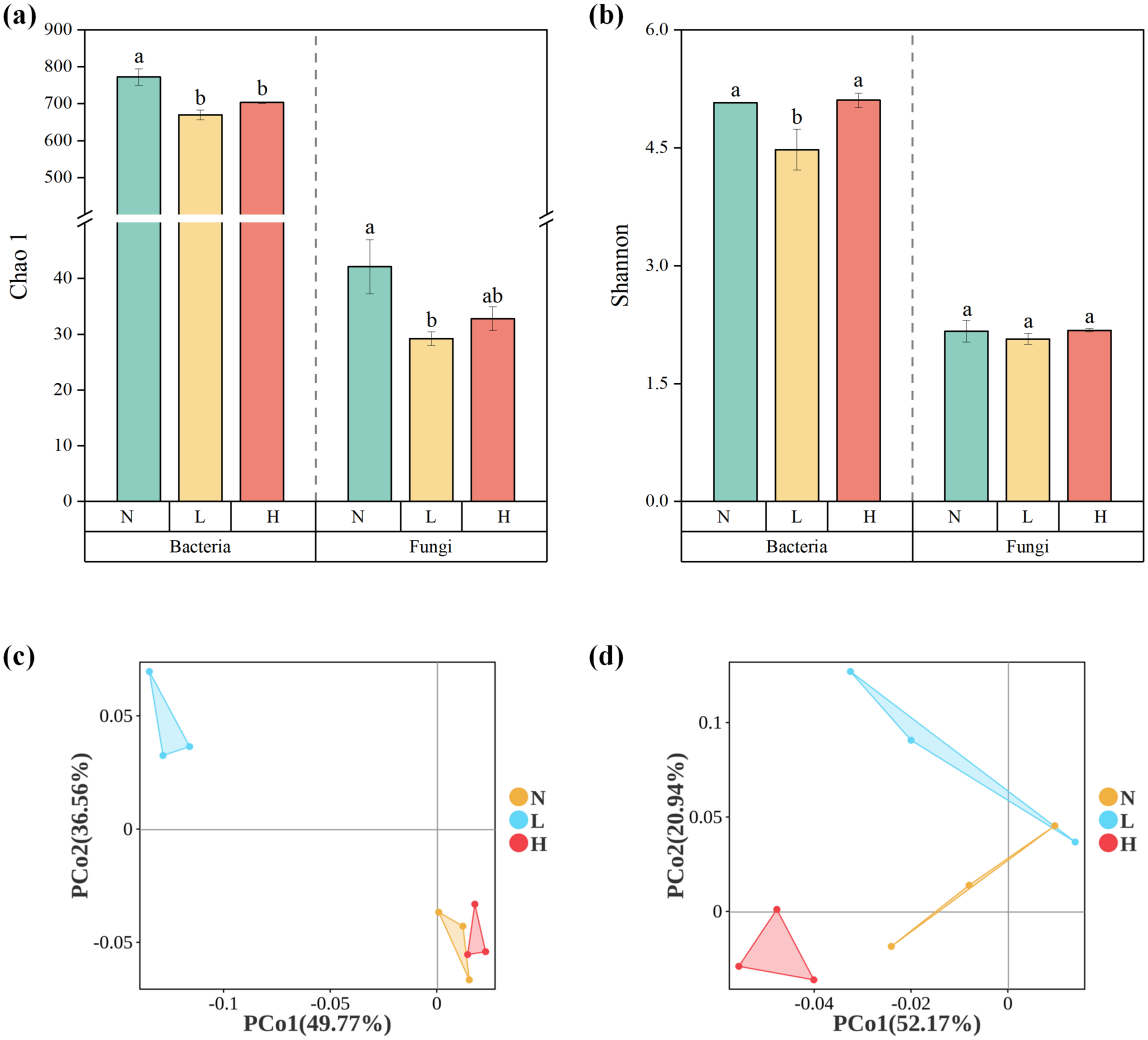
Diversity patterns of soil microbial communities under various expansion intensities of *E. jolkinii*: **(a)** *α*-diversity of bacterial communities; **(b)** α-diversity of fungal communities (Different letters above the bars indicate significant differences at *p* < 0.05). *β*-diversity of **(c)** bacterial and **(d)** fungal communities based on PCoA using Bray–Curtis dissimilarity. N: Soil in non-expanded patches by *E. jolkinii*; L: Soil in lightly expanded patches by *E. jolkinii*; H: Soil in heavily expanded patches by *E. jolkinii*.

The *E. jolkinii* was associated with clear increases in microbial diversity. In the bacterial community, both the Chao1 and Shannon indices were significantly lower in L patches than in N patches, while the Chao1 index was significantly reduced in H patches (*p <* 0.05; Figure 3a). In the fungal community, the Chao1 index in L patches was significantly lower than that in N patches (*p <* 0.05; Figure 3b). These results indicate that the expansion of *E. jolkinii* reduced the *α*-diversity of both soil bacterial and fungal communities.

The PCoA revealed relatively low within-group variation in soil bacterial communities across patch types. The bacterial communities of N and H patches were relatively similar, whereas the L patches were clearly separated from both N and H patches. In contrast, fungal communities exhibited greater within-group variation in N and L patches, together with pronounced differences among patch types (*p <* 0.05; Figures 3c,d). Overall, these findings suggest that the expansion of *E. jolkinii* substantially altered the composition and differentiation of microbial communities in montane meadow soils.

### 3.4 Impact of *Euphorbia jolkinii* expansion on soil carbon cycle functional genes

**Figure 4:**
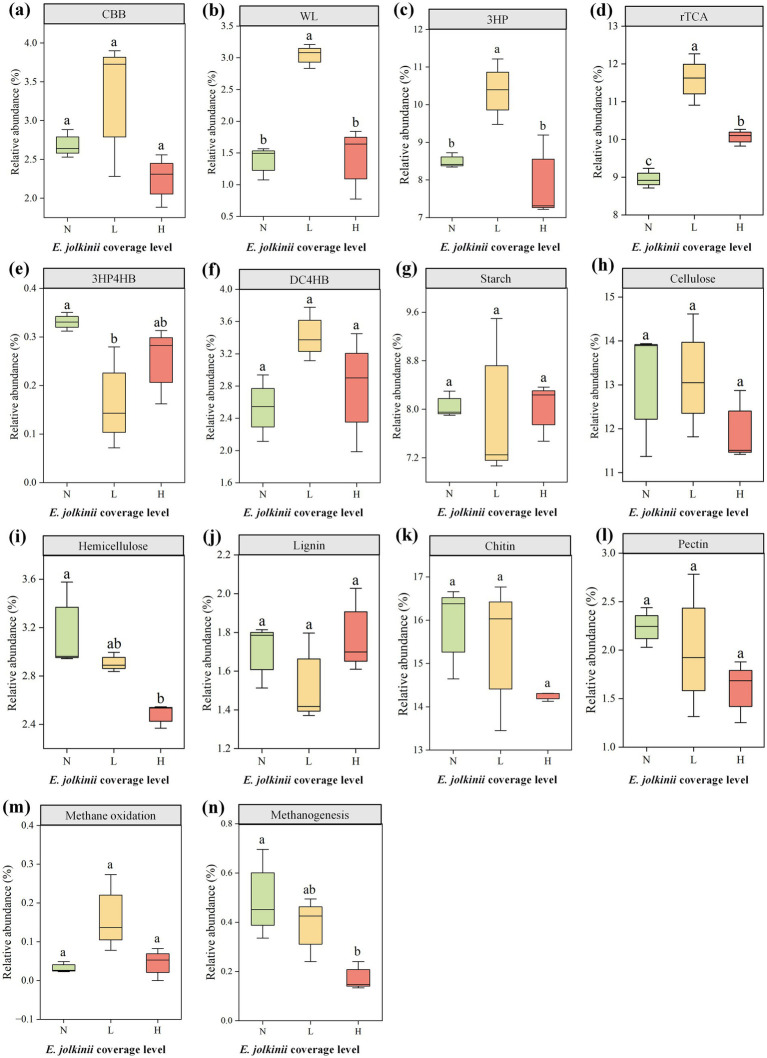
Variation in relative abundance of functional genes involved in soil carbon cycle across different patch types: **(a–f)** Functional genes associated with carbon fixation pathways; **(g–l)** Functional genes involved in carbon decomposition; **(m,n)** Functional genes related to methane metabolism [CBB, Calvin-Benson-Bassham cycle (reductive pentose phosphate pathway); rTCA, reductive tricarboxylic acid cycle; WL, Wood-Ljungdahl pathway (reductive acetyl-CoA pathway); 3HP, 3-hydroxypropionate bi-cycle; 3HP/4HB, 3-hydroxypropionate/4-hydroxybutyrate cycle; DC/4HB, dicarboxylate/4-hydroxybutyrate cycle]. Different letters above the boxplots indicate significant differences (*p* < 0.05). xpanded patches by *E. jolkinii*; L: Soil in lightly expanded patches by *E. jolkinii*; H: Soil in heavily expanded patches by *E. jolkinii*.

In subalpine meadows on the Southern Tibetan Plateau, carbon fixation was primarily mediated through the 3-hydroxypropionate bi-cycle (3HP) and the reductive tricarboxylic acid cycle (rTCA), followed by the Calvin-Benson-Bassham (CBB) cycle, the Wood-Ljungdahl (WL) pathway, the dicarboxylate/4-hydroxybutyrate (DC/4HB) cycle, and the 3-hydroxypropionate/4-hydroxybutyrate (3HP/4HB) cycle (Figures 4a–f). Compared with N patches, L patches exhibited significantly higher abundances of carbon fixation genes associated with the WL, 3HP, and rTCA pathways, whereas genes related to the 3HP/4HB pathway decreased significantly (*p <* 0.05). In H patches, only the abundance of rTCA-associated carbon fixation genes increased significantly, while no significant differences were observed for the remaining pathways. Carbon decomposition was dominated by chitin and cellulose degradation pathways, followed by starch, hemicellulose, pectin, and lignin degradation (Figures 4g–l). Relative to N patches, no significant changes in carbon decomposition gene abundance were detected in L patches, whereas genes associated with hemicellulose degradation were significantly reduced in H patches (*p <* 0.05). Methane metabolism was mainly represented by methane oxidation and methanogenesis pathways. Compared with N patches, no significant differences in methane metabolism gene abundance were observed in L patches, whereas genes involved in methanogenesis were significantly reduced in H patches (Figures 4m,n).

**Figure 5:**
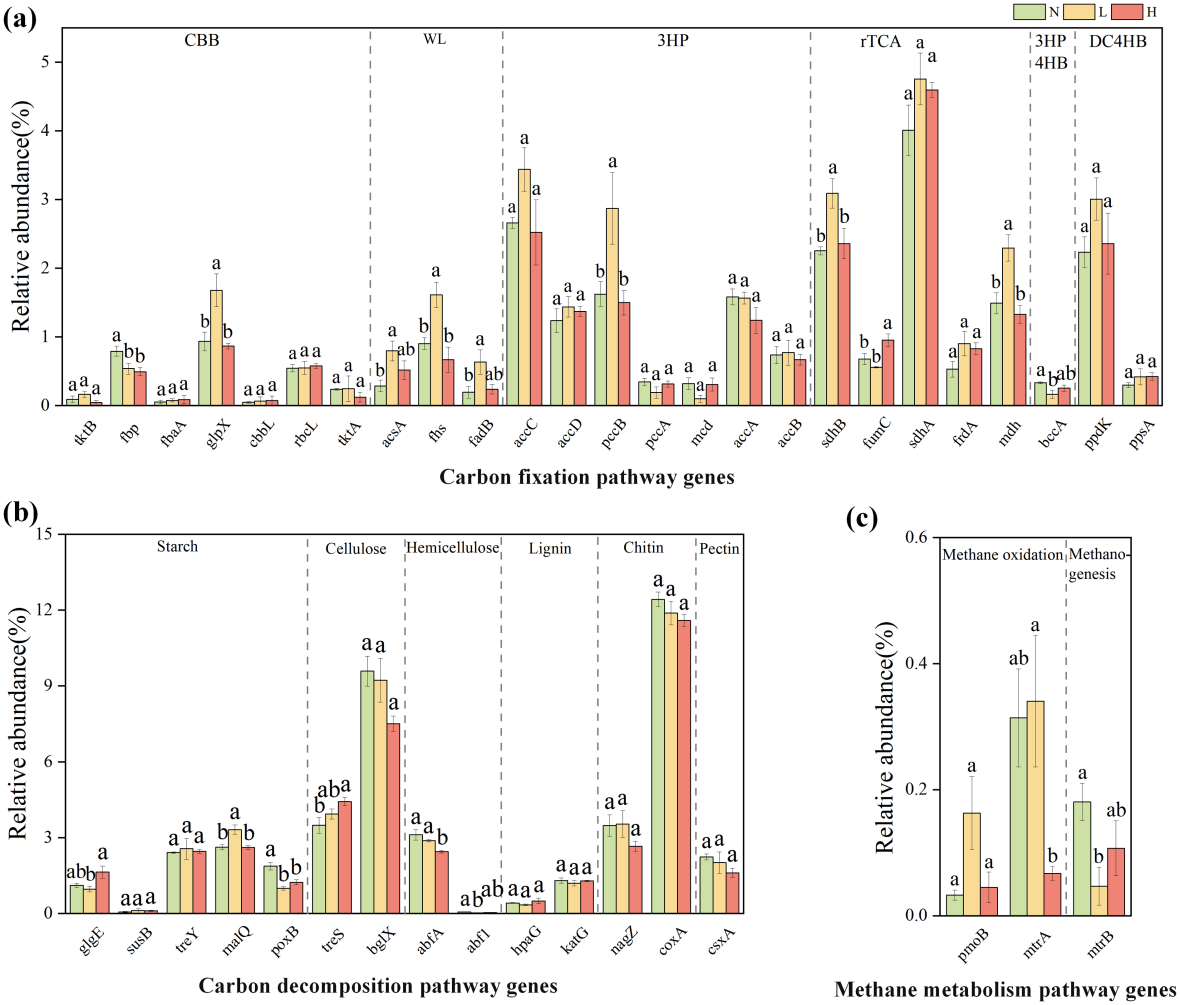
Abundance patterns of key functional genes involved in soil carbon cycle across different patch types: **(a)** Genes associated with carbon fixation pathways; **(b)** Genes involved in carbon decomposition processes; **(c)** Genes related to methane metabolism [Different letters above the bars indicate significant differences (*p* < 0.05)]. N: Soil in non-expanded patches by *E. jolkinii*; L: Soil in lightly expanded patches by *E. jolkinii*; H: Soil in heavily expanded patches by *E. jolkinii*.

KEGG-based annotation identified a total of 25 genes related to carbon fixation, 14 associated with carbon decomposition, and 3 involved in methane metabolism (Figures 5a–c). Among the carbon fixation genes, nearly half showed increased abundance coinciding with *E. jolkinii* expansion. Specifically, 8 genes were significantly enriched in both L and H patches compared with N patches (*p* < 0.05). These genes were distributed across several major fixation pathways. These included *glpX* involved in the CBB cycle; *acsA*, *fhs*, and *fadB* associated with the WL pathway; *pccB* related to the 3HP cycle; and *sdhB*, *fumC*, and *mdh* participating in the rTCA cycle (*p <* 0.05; Figure 5a). Among the 14 genes associated with carbon decomposition, only two genes exhibited significant enrichment in L or H patches (*p <* 0.05), namely *malQ*, involved in starch degradation, and *treS*, associated with cellulose degradation (*p <* 0.05; Figure 5b). In contrast, no significantly upregulated genes related to methane metabolism were detected in either L or H patches compared with N patches (Figure 5c).

### 3.5 Relationship among soil carbon cycle functional genes and physicochemical properties under *Euphorbia jolkinii* expansion

**Figure 6:**
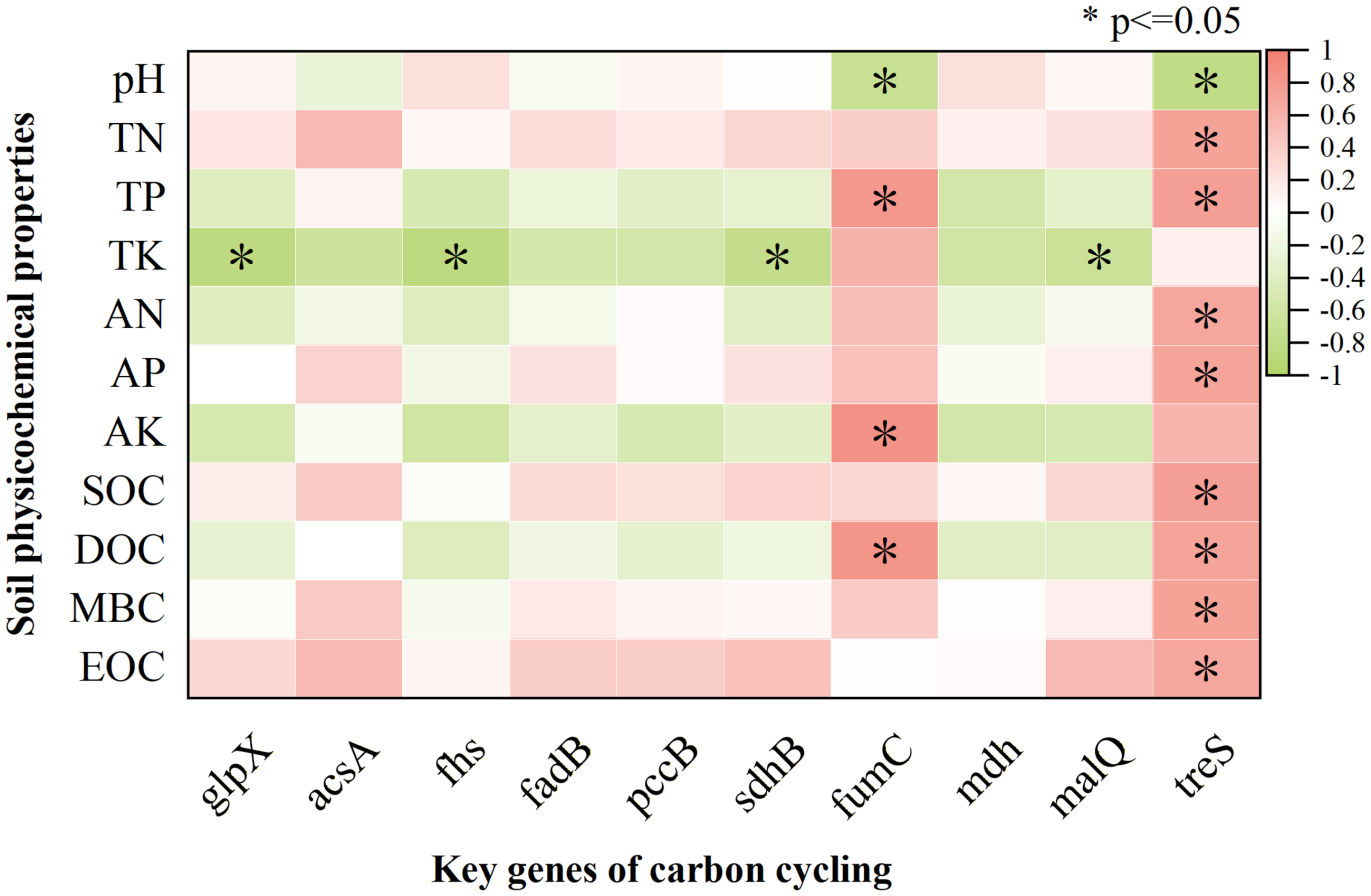
Relationships among key soil carbon cycle genes and soil properties under *E. jolkinii.*

Relationships between the 10 significantly enriched genes and soil physicochemical properties were further explored through correlation analysis. The results showed that *fumC* and *treS* were significantly negatively correlated with soil pH, whereas *glpX*, *fhs*, *sdhB*, and *malQ* showed significant negative correlations with soil TK content. In contrast, *fumC* was significantly positively correlated with TP, AK, and DOC contents, while *treS* exhibited significant positive correlations with TN, TP, AN, AP, SOC, DOC, MBC, and EOC contents (*p <* 0.05; Figure 6).

### 3.6 Factors influencing soil carbon cycle in subalpine meadows under *Euphorbia jolkinii*

**Figure 7:**
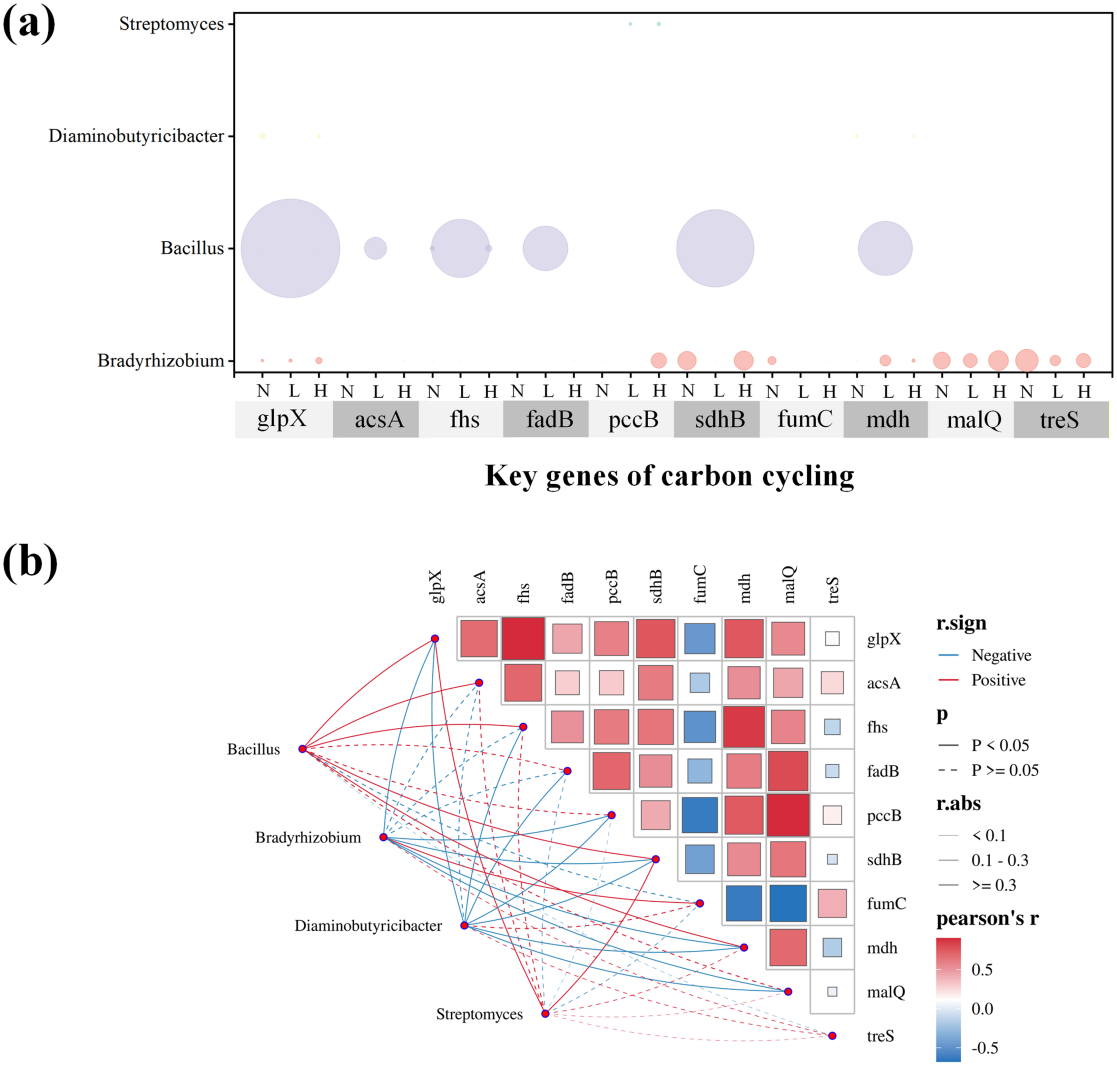
Distribution of carbon cycle genes across bacterial genera and their associations under *E. jolkinii* expansion: **(a)** Distribution of carbon fixation and decomposition genes among four bacterial genera across different patch types; Relationships between key **(b)** Correlations between key carbon fixation and decomposition genes and bacterial genera.

KEGG annotation revealed that the 10 carbon-cycling genes showing significant higher abundance coinciding with *E. jolkinii* expansion were primarily associated with four bacterial genera: *Bradyrhizobium*, *Bacillus*, *Diaminobutyricibacter*, and *Paraburkholderia* (Figure 7a). Correlation analyses between carbon fixation/decomposition genes and these bacterial genera further demonstrated that *glpX*, *acsA*, *fhs*, *sdhB*, and *mdh* were significantly positively correlated with *Bacillus*, whereas *fumC* showed a significant positive association with *Bradyrhizobium*. In addition, *glpX* and *sdhB* were significantly positively correlated with *Streptomyces* (*p <* 0.05; Figure 7b). Based on these relationships, *Bacillus*, *Bradyrhizobium*, and *Streptomyces* were selected for subsequent random forest analysis.

**Figure 8:**
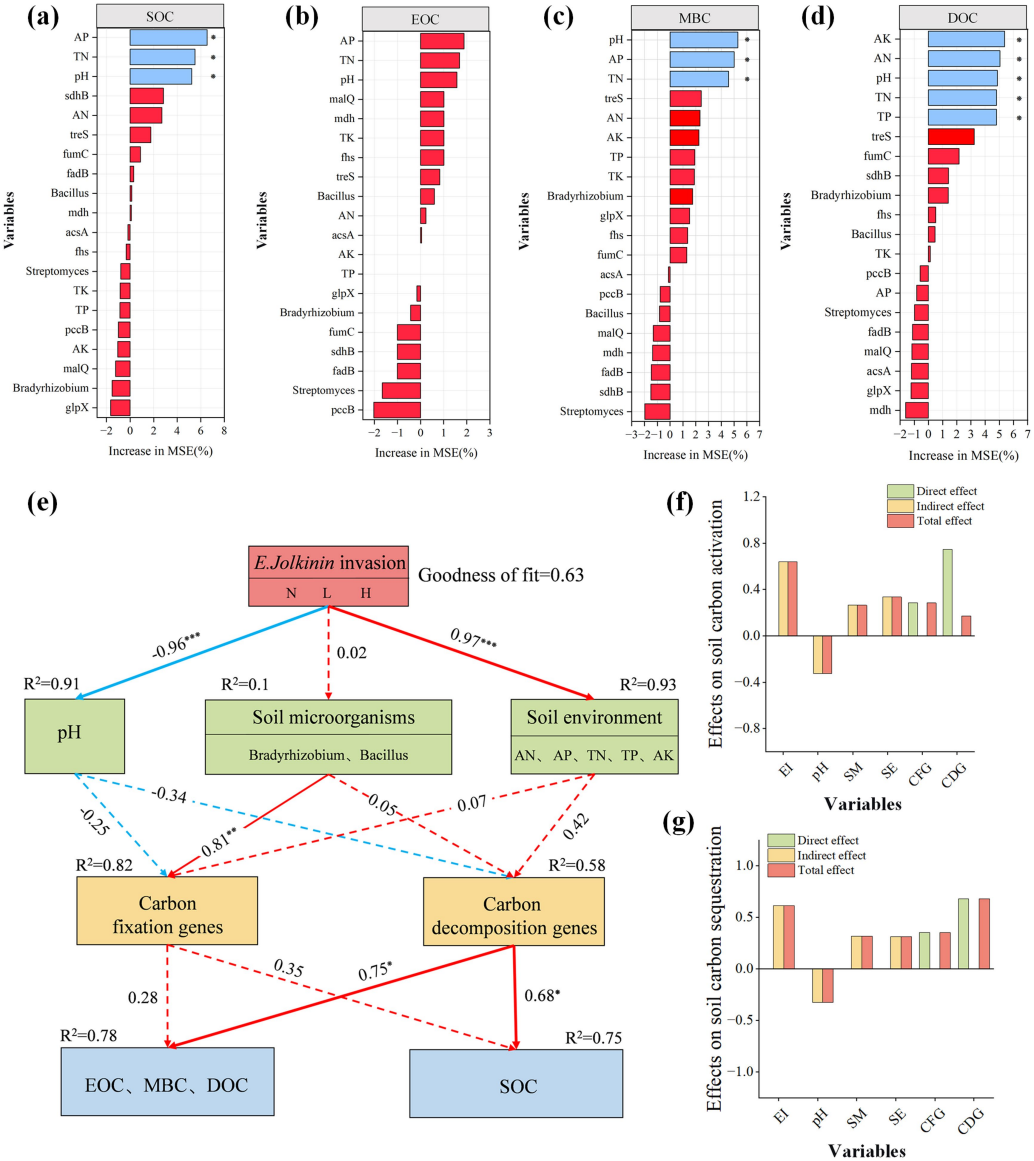
Key drivers of soil carbon fractions identified by random forest and PLS-PM analyses under *E. jolkinii*: Random forest results showing relative importance of soil environmental factors, microbial communities, and carbon cycle genes in influencing **(a)** SOC, **(b)** EOC, **(c)** MBC, and **(d)** DOC; Results of PLS-PM: **(e)** Path diagram illustrating relationships among variables [red and blue arrows indicate significant positive and negative effects, respectively (*p < 0.05, **p < 0.01, ***p < 0.001), while dashed arrows denote non-significant paths; numbers on the arrows represent path coefficients, and R^2^ indicates the explained variance]; **(f)** Direct, indirect, and total effects of EI (*E. jolkinii*), SM (soil microorganisms), SE (soil environment), CFG (carbon fixation genes) and CDG(carbon decomposition genes) on labile carbon fractions (EOC, MBC, DOC); **(g)** Direct, indirect, and total effects of the same factors on SOC.

Random forest analysis was conducted to identify the most important predictors associated with soil carbon fractions. The results indicated that soil environmental variables, including pH, AN, AP, TN, TP, and AK, together with the core microbial taxa *Bradyrhizobium* and *Bacillus*, showed high feature importance in relation to carbon “fixation” and “transformation” coinciding with *E. jolkinii* expansion. In addition, several carbon fixation genes (*acsA*, *fhs*, *fumC*, *fadB*, *sdhB*, and *mdh*) and carbon decomposition genes (*malQ* and *treS*) were identified as key functional factors linked to soil carbon dynamics in montane meadow ecosystems (Figures 8a–d).

Building on these results, PLS-PM was constructed, yielding a GOF of 0.63 and indicating strong explanatory capacity (Figures 8e–g). The model revealed that the expansion of *E. jolkinii* was linked to soil carbon dynamics primarily in relation to variations in soil environmental conditions and microbial communities. Specifically, soil pH exerted negative path coefficients on key genes involved in carbon fixation and decomposition, whereas microbial communities and soil nutrient variables, including AN, AP, TN, TP, and AK, showed positive associations with these functional genes. Among these factors, microbial communities showed the strongest positive relationship with carbon fixation processes. Both carbon fixation and carbon decomposition genes coincided with higher soil carbon accumulation (Figure 7e). Total effect analysis further demonstrated that soil nutrient variables (AN, AP, TN, TP, and AK) had the greatest overall total effect on EOC, MBC, and DOC contents, whereas carbon decomposition genes exerted the strongest total effect on SOC (Figures 8f–g).

## 4 Discussion

### 4.1 *Euphorbia jolkinii* gains a competitive advantage through soil acidification and increased nutrient availability

Poisonous weeds often achieve ecological expansion by modifying soil environments and thereby gaining advantages in nutrient acquisition (Abbas et al., 2023). The patterns observed here suggest that *E. jolkinii* follows a similar strategy. A consistent decline in soil pH was detected in expanded patches, reflecting a potential relationship with organic acid secretion from roots and the release of acidic compounds during litter decomposition. Similar patterns have previously been reported for *S. chamaejasme* (Zhang et al., 2021). Soil organic matter represents a major reservoir and turnover pool for nutrient cycling in terrestrial ecosystems (Farooqi et al., 2024). *E. jolkinii* expansion was associated with markedly higher soil nitrogen, phosphorus, and carbon fractions, consistent with earlier findings that poisonous weeds coincide with localized “fertility islands” (Xue et al., 2025). These results suggest that nutrient enrichment may constitute an important ecological strategy associated with the expansion of poisonous weeds in grassland ecosystems. Notably, soil TK did not differ significantly among patch types, whereas AK was substantially higher in heavily expanded patches. This pattern indicates that *E. jolkinii* expansion is associated with the activation of mineral-bound potassium rather than altering the overall potassium pool. Because most soil potassium is structurally bound within primary minerals and generally remains stable over short time scales (Ayangbenro and Babalola, 2021), the observed increase in AK is likely linked to root-induced mineral weathering. Organic acids released by roots, such as citric acid and oxalic acid, may accelerate the dissolution of potassium-bearing minerals including mica and feldspar (Awoniyi et al., 2025; Upadhyay et al., 2022). In addition, root exudates may improve rhizosphere conditions and stimulate microbial activity, which is potentially linked to the mobilization and release of insoluble potassium and ultimately coincides with higher soil AK availability (Yu et al., 2026).

The expansion of *E. jolkinii* coincides with the activation of soil carbon fractions, reflecting a soil carbon environment associated with its own growth. As a central component of the soil carbon pool (Zhao et al., 2024), SOC was significantly higher in both L and H patches; this variation was potentially linked to the species’ well-developed root system and high aboveground biomass, which may contribute substantial inputs of plant residues and root exudates to the soil (Wen et al., 2022). Meanwhile, *E. jolkinii* encroachment was accompanied by increased soil nitrogen and phosphorus availability; this nutrient enrichment co-occurred with optimized microbial metabolic conditions and a greater stability and retention capacity of SOC. These changes were associated with greater soil carbon accumulation (Qin et al., 2022). The pronounced increases in labile carbon fractions, including EOC, MBC, and DOC, further demonstrate that *E. jolkinii* expansion is associated with the priming and activation of soil carbon cycling processes.

Among these components, EOC, characterized by its rapid transformation, is widely recognized as a sensitive indicator of carbon pool dynamics (Xiao W. et al., 2025; Xiao X. et al., 2025; Xiao Y. et al., 2025). The observed increase in EOC within expanded patches suggests that a larger proportion of carbon exists in more reactive and bioavailable forms, thereby supporting microbial metabolism. MBC exhibited a similar upward trend. As an indicator closely associated with soil quality (Lagomarsino et al., 2008), higher MBC values imply that enhanced carbon availability promotes microbial proliferation. This increase in microbial biomass likely accelerates the transformation of organic carbon, contributing to a reinforcing feedback loop in which carbon inputs stimulate microbial activity and, in turn, enhance the accumulation of labile carbon fractions. In contrast, DOC, which serves as an important energy source for microorganisms and is highly mobile in soil systems (Yu et al., 2019). In this study, DOC increased significantly only in L patches, whereas no significant change was observed in H patches. This pattern may reflect a variation in the balance between carbon input and decomposition rates along the expansion gradient. In lightly expanded patches, carbon inputs derived from root exudates and litter potentially exceeded microbial decomposition and leaching losses, coinciding with DOC accumulation (Hao et al., 2024). In contrast, the greater availability of organic substrates in heavily expanded patches was associated with higher microbial biomass and activity, which relates to a faster DOC turnover and decomposition (Liu et al., 2025). At the same time, intensified competition for carbon uptake by *E. jolkinii* may be linked to the restriction of DOC accumulation, ultimately leading to a relatively stable equilibrium.

### 4.2 *Euphorbia jolkinii* promotes population expansion by enriching acid-tolerant functional microorganisms and activating recalcitrant soil carbon

Soil microorganisms function as key decomposers in terrestrial ecosystems, playing an essential role in material turnover and energy flow, and thereby contributing to the stability of ecosystem structure and function (Wang et al., 2024). The introduction and spread of toxic weeds can disrupt these processes by reshaping microbial community composition and, consequently, altering ecosystem functioning (Kama et al., 2023). In this study, the expansion of *E. jolkinii* was associated with marked variations in soil microbial community structure, likely in relation to changes in soil pH and nutrient availability. Within the bacterial community, oligotrophic taxa, including *Bradyrhizobium*, *Diaminobutyricibacter*, and *Edaphobacter*, declined, whereas copiotrophic genera such as *Streptomyces* and *Bacillus* became enriched. This shift suggests a potential transition in microbial ecological strategies from oligotrophy to copiotrophy coinciding with increased nutrient availability. Similar patterns have been reported during the expansion of *Veratrum nigrum* in degraded grasslands (Pang et al., 2025). In addition, *Bradyrhizobium*, *Paraburkholderia*, and *Edaphobacter* were negatively correlated with soil pH (Supplementary Table 1), indicating that these acid-tolerant microorganisms may possess competitive advantages under acidic soil conditions (Zhou et al., 2023). Within the fungal community, the increased abundance of *Rhizopus* in lightly expanded patches and *Aspergillus* in heavily expanded patches suggests that *E. jolkinii* expansion correlates with the enrichment of fungal taxa with specific ecological functions, which accompanies variations in rhizosphere organic matter decomposition and carbon turnover (Liu et al., 2025).

The expansion of *E. jolkinii* was associated with a significant reduction in the *α*-diversity of both bacterial and fungal communities, accompanied by pronounced microbial community differentiation. These findings coincide with previous reports showing a lower soil microbial α-diversity in relation to *S. chamaejasme* expansion in alpine grasslands (Ding et al., 2026). The decline in microbial diversity is potentially linked to soil acidification and the release of allelochemicals co-occurring with expansion, processes that may selectively correlate with the enrichment of specialized functional taxa while exhibiting a negative relationship with the growth of a broader range of microorganisms (Xiao et al., 2026). Furthermore, PCoA revealed distinct variations in the *β*-diversity of bacterial and fungal communities across different *E. jolkinii* expansion patches, suggesting substantial restructuring of microbial community composition in montane meadow soils.

### 4.3 *Euphorbia jolkinii* improves soil carbon availability and facilitates its expansion by enriching carbon-cycling microbial communities and their associated functional genes

The structure of soil microbial communities and the abundance of carbon-cycling functional genes are key biological regulators of soil carbon turnover (Liao et al., 2022). In the present study, the microbial genera *Bradyrhizobium* and *Bacillus*, together with carbon fixation genes (*acsA*, *fhs*, *fumC*, *fadB*, *sdhB*, and *mdh*) and carbon decomposition genes (*malQ* and *treS*), were closely associated with soil carbon fixation and transformation in montane meadows undergoing *E. jolkinii* expansion (Figure 8). Among these taxa, *Bradyrhizobium* actively participates in key carbon fixation pathways, and its presence coincides with the conversion of inorganic carbon into organic forms, which is linked to variations in soil carbon storage (Shan et al., 2025). In contrast, *Bacillus* exhibits strong organic matter degradation capabilities, reflecting a potential role in the decomposition and transformation of complex carbohydrates, which may modulate the turnover of labile soil carbon pools (Chen et al., 2026). The enrichment of these functional microbial groups provides a biological basis for soil carbon fixation and transformation, representing an important microbial profile associated with *E. jolkinii* expansion and its correlation with soil carbon cycling.

The abundance of microbial carbon fixation genes reflects the carbon sequestration potential of microbial communities, with higher gene abundance generally indicating a greater capacity for carbon fixation (Xiao et al., 2024; Yuan et al., 2024). Previous studies have identified multiple carbon fixation pathways, including the rTCA cycle, WL pathway, DC/4-HB cycle, 3-HP bicycle, 3-HP/4-HB cycle, and the Calvin cycle, while carbon decomposition is primarily associated with the decomposition of complex carbohydrates such as starch, hemicellulose, cellulose, chitin, pectin, and lignin (Huang et al., 2022; Huang et al., 2025). In this study, genes involved in the 3HP and rTCA pathways showed the highest abundance among all carbon fixation pathways. Several genes, including *acsA*, *fhs*, *fumC*, *fadB*, *sdhB*, and *mdh*, were identified as key components of microbial carbon fixation processes, and their elevated abundance in expanded patches is associated with microbial carbon metabolic activity in meadow soils (Xiao et al., 2025; Xiao et al., 2025; Xiao et al., 2025; Chen et al., 2026). Carbon decomposition processes exhibited a similar acceleration. Microbial-mediated decomposition is a central mechanism driving organic carbon turnover (Naylor et al., 2020). In the present study, key genes responsible for the degradation of cellulose, starch, hemicellulose, chitin, and pectin, particularly *malQ* and *treS*, were significantly enriched in *E. jolkinii* expansion patches, suggesting a potential shift in microbial capacity for complex organic carbon degradation in relation to *E. jolkinii* expansion. These functional variations were closely associated with the enrichment of core functional microbial genera, especially *Bradyrhizobium* and *Bacillus*, which collectively coincide with accelerated soil carbon transformation processes (Cao et al., 2025; Fan et al., 2022).

In summary, the expansion of *E. jolkinii* significantly reduced soil pH while increasing the contents of TN, AP, AN, TP, and AK in subalpine meadows soils. These alterations in the soil microenvironment may have driven shifts in microbial community structure and diversity, thereby regulating soil carbon fixation and decomposition processes and ultimately influencing soil carbon cycling and transformation. However, the present study cannot conclusively determine whether these changes were directly induced by *E. jolkinii* expansion or whether pre-existing differences in soil microenvironmental conditions initially facilitated its establishment. Therefore, future studies integrating long-term field monitoring and controlled experiments are needed to clarify the causal relationships between *E. jolkinii* expansion and soil carbon cycling dynamics.

## 5 Conclusion

*Euphorbia jolkinii* expansion was associated with significant alterations in soil properties, including a decline in pH and concurrent increases in nutrient availability (nitrogen, phosphorus, and potassium), as well as microbial diversity. These changes were accompanied by the establishment of a distinct soil microenvironment characterized by the dominance of key microbial genera such as *Bradyrhizobium* and *Bacillus*. Functionally, these microbial groups appear to act in a coordinated manner, in relation to intensified activity in both carbon fixation pathways (e.g., rTCA and 3-HP cycles) and carbon decomposition processes (notably chitin and cellulose decomposition). Concurrently, multiple soil carbon fractions, including SOC, EOC, MBC, and DOC, were elevated in expanded patches. Overall, the findings suggest that *E. jolkinii* expansion is associated with the restructuring of soil microbial communities and with accelerated carbon cycling processes, thereby coinciding with increased availability of organic carbon and the creation of soil conditions that may further support its persistence and expansion.

## Data Availability

The data presented in this study are publicly available. The data can be found here: https://ngdc.cncb.ac.cn/gsa/browse/CRA043642, accession number CRA043642. Further inquiries can be directed to the corresponding author.
