# Supplementary material for: Microbial feedback drives soil carbon fixation and nutrient transformation during Euphorbia jolkinii expansion in subalpine meadows
Source: Front Microbiol. 2026 Jul 15;17:1864391. doi: 10.3389/fmicb.2026.1864391 (PMC13416067; doi:10.3389/fmicb.2026.1864391)
Supplement: Supplementary file 1 [file Table_1.DOCX]

Supplementary Material

# Supplementary Tables

**Supplementary Table 1.** Basic information of sampling units.

| Patch type | N | L | H |
| --- | --- | --- | --- |
| Total aboveground biomass (g·DM·m^-2^) | 424.27 | 544.93 | 640.76 |
| *E. jolkinii* above ground biomass (g·DM·m^-2^) | 0 | 72.12 | 301.45 |
| *E. jolkinii* coverage (%) | 0 | 8.9 | 41.2 |
| *E. jolkinii* importance value | 0 | 7.81 | 28.75 |
| *P. crymophila* importance value. | 35.64 | 32.57 | 15.38 |
| Vegetation status | In N patch, *P. crymophila* serves as the monospecific dominant species. | Despite L patch, *P. crymophila* remains the dominant species | Under H patch, *E. jolkinii* becomes the dominant species, while *P. crymophila* is declines to a subdominant position |
| Remarks | The study area was enclosed in 2012 following a period of intensive grazing prior to fencing | | |

# Supplementary Figures


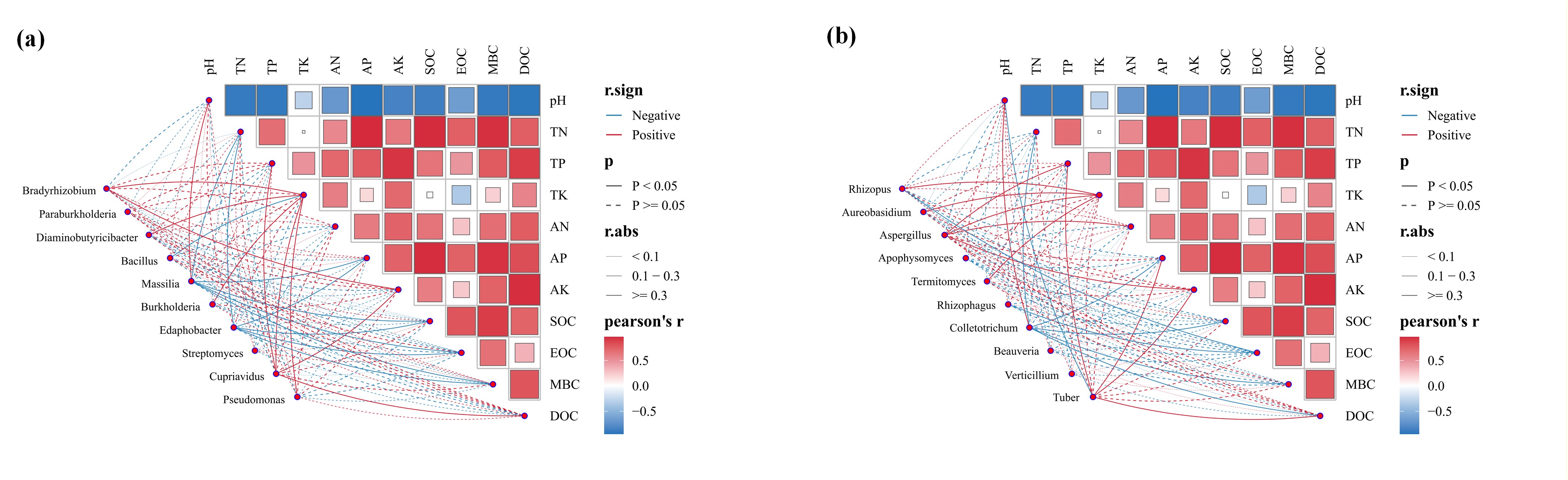


**Supplementary Figure 1.** Correlation analysis between soil physicochemical properties and microbial genera under EI: (a) Physicochemical properties and bacterial genera; (b) Physicochemical properties and fungal genera.
